# Case Report: Late-Presenting Congenital Diaphragmatic Hernia With Tension Gastrothorax

**DOI:** 10.3389/fped.2021.618596

**Published:** 2021-04-14

**Authors:** Aabha A. Anekar, Sumana Nanjundachar, Dhaneshgouda Desai, Jafferali Lakhani, Prakash M. Kabbur

**Affiliations:** Train and Help Babies Organization, Dallas, TX, United States

**Keywords:** congenital diaphragmatic hernia, tension gastrothorax, Bochdalek hernia, gastric volvulus, tension pneumothorax

## Abstract

A congenital diaphragmatic hernia (CDH) occurs when the abdominal contents protrude into the thoracic cavity through an opening in the diaphragm. The main pathology lies in the maldevelopment or defective fusion of the pleuroperitoneal membranes. Delayed diagnosis in later childhood as in the index case reported here can lead to life-threatening complications such as tension gastrothorax and gastric volvulus. Such life-threatening conditions should be managed emergently avoiding misdiagnoses and untoward harm to the patient. We report a pediatric case of an 8-year-old boy who presented with respiratory distress, chest pain, and non-bilious vomiting. He was initially diagnosed with tension pneumothorax, and the chest x-ray was interpreted as hydropneumothorax. A chest tube placement was planned but was withheld due to excessive vomiting. A nasogastric (NG) tube was placed, and a barium-filled radiograph showed an intrathoracic presence of the stomach. A diagnosis of a congenital diaphragmatic hernia with tension gastrothorax was made. The posterolateral (Bochdalek) diaphragmatic hernia was repaired successfully. This case report highlights the importance of including a late-presenting CDH in the differential diagnoses of pediatric patients who present with respiratory distress, chest pain, non-bilious vomiting, and radiological findings suggestive of tension pneumothorax.

## Introduction

A congenital diaphragmatic hernia (CDH) is a pathological condition diagnosed mainly in neonates with a survival rate of 67% (1). The incidence of CDH varies from 0.8 to 5 for every 10,000 births (2). The etiology is broadly categorized into genetic, environmental, and nutritional causes. It can be associated with many genetic syndromes with underlying chromosomal abnormalities such as Cornelia de Lange and Pallister-Killian syndromes. However, late-presenting CDH is usually an isolated finding and prone to rare complications such as tension gastrothorax, splenic rupture, or intestinal obstruction (3). The most recent literature search revealed four pediatric cases of tension gastrothorax associated with a late CDH and gastric volvulus (4). This report illustrates a case of delayed presentation of posterolateral diaphragmatic hernia complicated by tension gastrothorax with an initial clinical diagnosis of tension pneumothorax.

## Case Description

An 8-year-old male child presented to the pediatric outpatient clinic with a sudden onset of dyspnea and chest pain for 12 h. The child also complained of three episodes of non-bilious vomiting. He had no similar complaints in the past. As per the mother's history, the pregnancy, birth history, and postpartum period were uneventful. The pregnancy was in a remote village, and prenatal scans or documents were not available. The general examination showed a dehydrated patient with tachycardia (140 beats/min), mild hypotension (90/60 mmHg), and tachypnea (60 breaths/min). His oxygen saturation was 82% on room air. On auscultation, the breath sounds were absent on the left side of the chest with resonance elicited on percussion. The heart sounds were better heard on the right side, indicating a shift in the mediastinum. The abdomen was flat with normal bowel sounds. No bowel or gastric peristaltic sounds were heard in the left hemithorax. The patient was clinically diagnosed with tension pneumothorax.

**Figure 1:**
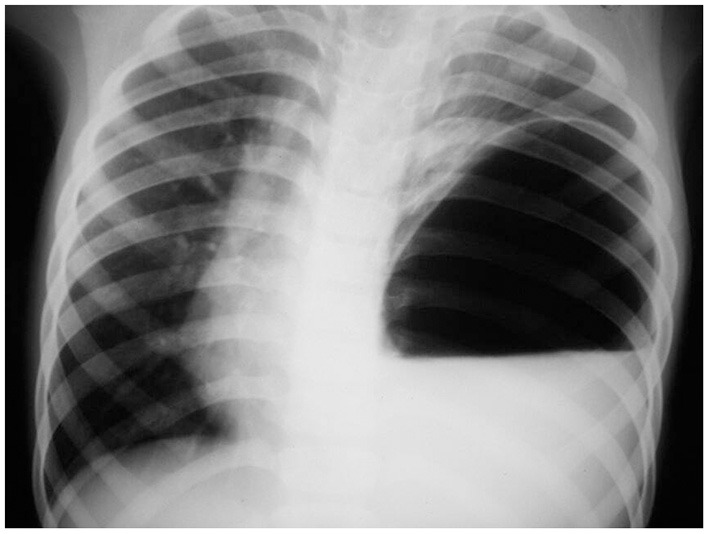
The plain chest x-ray shows an air-fluid level on the left side of the chest with an absent gastric shadow, initially interpreted as hydropneumothorax.

**Figure 2:**
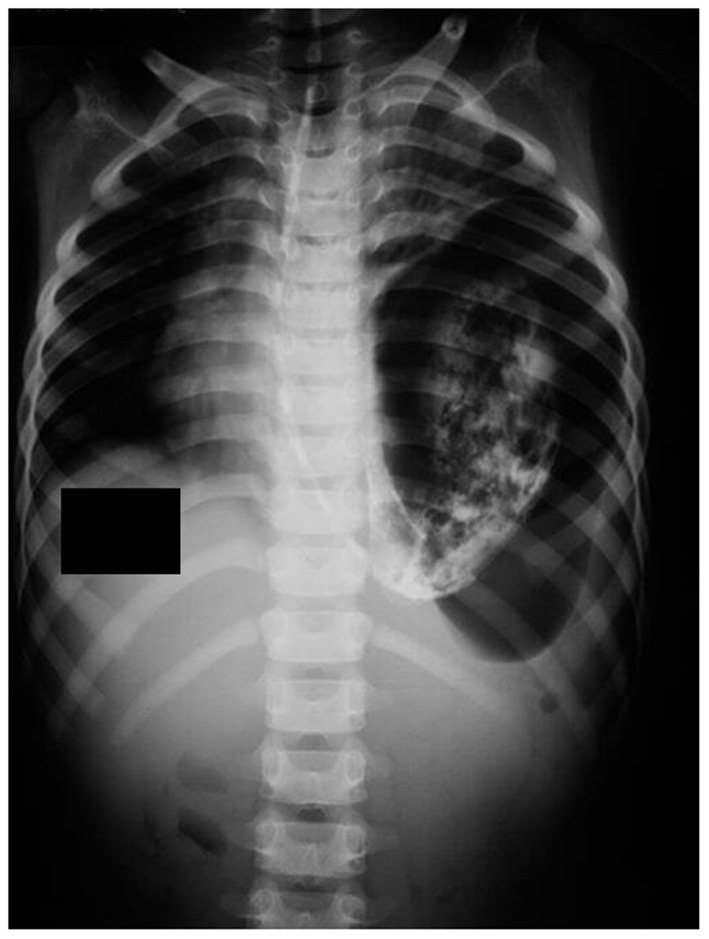
The barium-filled chest x-ray shows the NG tube and the contrast present in the thoracic cavity, indicating a CDH.

The x-ray showed an air-fluid level suggesting an encysted hydropneumothorax or pyopneumothorax with a mediastinal shift (Figure 1). Oxygen and IV fluids were administered. A chest tube drainage was planned based on the initial x-ray. Due to excessive vomiting, an NG tube was placed, and 400 mL of gastric fluid was aspirated. This large amount of gastric fluid prompted another x-ray, which showed the NG tube in the thoracic cavity with a single large air bubble on the left side. The lab investigations showed a hemoglobin—9.4 g/dL, leukocyte count—7,600 cells/mm^3^ (N-74%, L-22%, E-4%), random blood sugar—98 mg/dL, serum sodium—136 mEq/L, serum potassium—3.8 mEq/L, serum creatinine−0.8 mg/dL. Since the patient presented in a resource-poor area, a CT scan could not be obtained. Instead, a barium study was done with an NG tube, which showed a CDH with tension gastrothorax (Figure 2).

**Figure 3:**
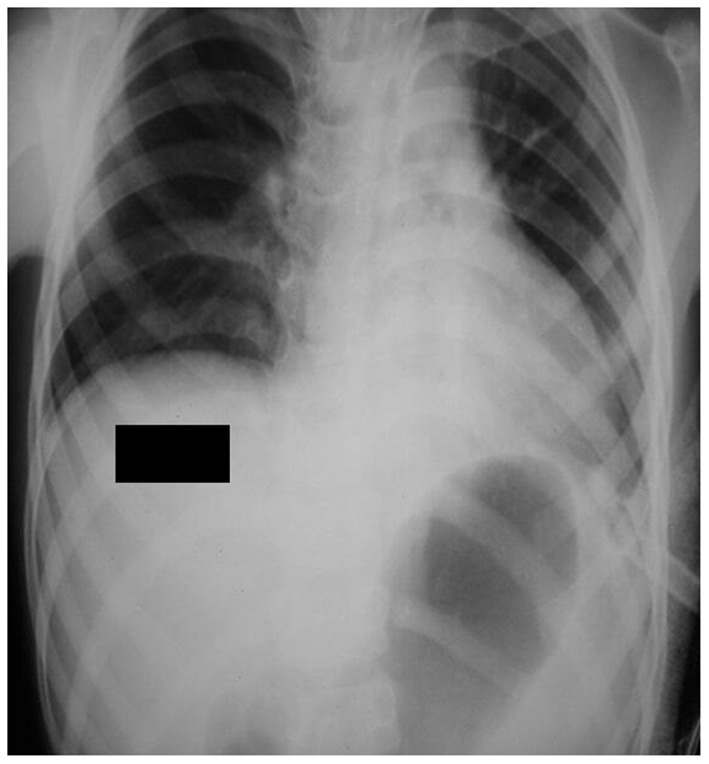
The follow-up chest x-ray shows a normal mediastinum with the gastric bubble present below the diaphragm.

An emergency laparotomy was done, which revealed a left posterolateral diaphragmatic defect through which the stomach, spleen, small bowel loops, and colon herniated into the left thorax (Figure 3). The stomach was grossly dilated with one organo-axial turn, and the left lung was compressed. All organs were viable and were returned to the abdominal cavity, and the diaphragmatic defects were closed in two layers along with gastropexy.

The postoperative x-ray was normal (Figure 3). The patient recovered within the following 2 days and was discharged on postoperative day 5. The patient was followed up to a year after surgery and he did not report any history of vomiting, abdominal pain, or respiratory distress. He had a satisfactory weight gain of 5 kg during this period. The patient had a favorable prognosis due to the presence of isolated CDH with no other associated congenital abnormalities.

## Discussion

A congenital diaphragmatic hernia (CDH) is characterized by the protrusion of abdominal contents into the thoracic cavity through an opening in the diaphragm. A prenatal ultrasound can diagnose a CDH only when the defect is obvious or the abdominal contents have herniated into the thoracic cavity (5). When the condition goes undiagnosed during the antenatal period, it is usually detected within 1 month after birth. However, about 10% of CDH patients are diagnosed later in life (6). These patients usually have a normal chest x-ray (taken for other reasons) before the presentation. Increased intra-abdominal pressure can push the abdominal organs into the thoracic cavity through an existing defect, which can be seen on the chest x-ray (7). Berman et al. describe 26 cases of late CDH that previously had a normal chest x-ray, suggesting a combination of congenital and acquired factors responsible for the late presentation (8).

When the stomach protrudes into the thoracic cavity, it can become massively distended with air and/or fluid in a closed-loop, resulting in tension gastrothorax, an extremely rare, life-threatening condition. The distended stomach can compress on the lung, leading to severe respiratory distress, reduced or absent breath sounds, and a shift in the mediastinum, much like a tension pneumothorax. Horst et al. describe a case series of five children with tension gastrothorax. Only one out of these five children were correctly diagnosed with tension gastrothorax (9). Bagłaj and Dorobisz show in their review that out of 349 children with a late-presenting posterolateral hernia, 88 of them (25.2%) were initially diagnosed as pneumothorax or pleural effusion based on the radiographic findings (10). The inadvertent placement of the chest tube in such situations will perforate the organs leading to dreaded complications such as sepsis, bowel perforation, acute lung injury, and respiratory failure, all accompanied by increased morbidity and mortality (11). Hence, it is crucial to differentiate between these two conditions to determine the further course of management and prevent additional complications. While CT scan is superior in evaluating co-existing abnormalities, chest x-ray takes precedence in acute, unstable patients and resource-poor settings (12). Table 1 shows the differences in the radiographic findings between tension pneumothorax and tension gastrothorax (13).

**Table 1:** The differences in the x-ray findings between tension pneumothorax and tension gastrothorax.

	**Tension gastrothorax**	**Tension pneumothorax**
Description	Large air bubble compressing the lung	Compressed lung central in position with surrounding intrapleural air
Hemidiaphragm	Ill-defined or elevated	Clearly marked
Gastric bubble	Not seen in the abdominal cavity	Present in the abdominal cavity
Position of NG tube	In the thoracic cavity	In the abdominal cavity

*Radiographic Findings (X-Ray) of Tension Gastrothorax vs. Tension Pneumothorax*.

The mainstay of management of tension gastrothorax is the timely decompression of the stomach, without which the mediastinal shift can impair venous return and cause cardiovascular collapse. Okuda et al. describe a case of late CDH with acute tension gastrothorax where the patient presented with cardiopulmonary arrest and failed resuscitation (4). The NG tube should be placed immediately at presentation, although an aggressive and forceful placement can lead to perforation, especially in children (14, 15). An endoscopic approach could be tried in stable patients. If all of the above interventions are unsuccessful, emergent thoracotomy or laparotomy is indicated (16). The type of approach depends on the surgeon's preference, any associated abdominal organ involvement, and the patient's condition (16, 17). In the index case, the laparotomy approach was preferred due to the presence of gastric volvulus.

An acute gastric volvulus is a surgical emergency, as the non-operative mortality is as high as 80% (18). The repair of an acute gastric volvulus involves the following components:

- Decompression of the stomach
- Reduction of the volvulus
- Fixation of the stomach (gastropexy)
- Treating the underlying cause.

The laparoscopic approach is gaining more popularity due to its associated early recovery and reduced length of hospital stay (18, 19). Yates et al. presented a case series in which 11 patients underwent laparoscopic repair of gastric volvulus with a relief of obstructive symptoms and no recurrence (20). However, laparotomy remains the preferred choice in low and middle-income countries. The underlying cause of the complications was a Bochdalek hernia in the index case presented here, which was closed in two layers successfully.

## Conclusion

Our case illustrates the importance of considering a late-onset CDH with tension gastrothorax when a pediatric patient presents with respiratory distress and abdominal symptoms, such as vomiting and dehydration. It is important to insert an NG tube, then get an x-ray of the chest, along with proper identification of the tip position. If the tip is in the thoracic cavity, a further emergent workup is warranted along with the transfer to a pediatric surgical facility for the successful management of associated complications.

Our case report focuses on the management of tension gastrothorax in a resource-limited setting. The choice of investigation and type of surgery were tailored according to the availability of resources. The successful management of the condition even in the absence of optimal technology can help guide clinicians and surgeons in low and middle-income countries. Since cases similar to our index case often present to the outpatient pediatric clinics and emergency departments, this report is useful for the first-line responders on how to identify life-threatening cases of CDH and tension gastrothorax.

## Patient Perspective

The patient was initially very scared because of the severity of his symptoms. He felt a little uncomfortable undergoing the necessary procedures. After the surgery, he had moderate, dull-aching pain around the surgical area for about 2 weeks. However, the pain subsided and his appetite and activity level gradually improved over the next month. After a year from the presentation, he had a 5-kg weight gain and felt more energetic. He did not report any similar pulmonary or gastrointestinal symptoms thereafter.

## Data Availability Statement

The original contributions presented in the study are included in the article/Supplementary Material, further inquiries can be directed to the corresponding author/s.

## Author Contributions

JL, PK, and SN were responsible for the conception of the idea of the case report. DD, JL, and PK contributed to the data collection. AA prepared the first draft of the manuscript. PK wrote a few sections of the report. All the authors contributed to the revision and submission of the manuscript.

## Conflict of Interest

The authors declare that the research was conducted in the absence of any commercial or financial relationships that could be construed as a potential conflict of interest.
