# Supplementary material for: Case Report: Late-Presenting Congenital Diaphragmatic Hernia With Tension Gastrothorax
Source: Front Pediatr. 2021 Apr 14;9:618596. doi: 10.3389/fped.2021.618596 (PMC8081028; doi:10.3389/fped.2021.618596)
Supplement: Supplementary file 1 [file Table_1.DOCX]

Supplementary Material

# Supplementary Data

The literature included in the discussion part of our case report includes patients with different ages, clinical presentation, investigations, and treatment. The tables below compare these differences of the referenced cases with our case. Diaphragmatic hernias in children are usually congenital, though acquired factors can also be present, whereas in adults, the cause is mainly trauma-related. However, the approach to the condition and its complications remains the same, regardless of the age of the patient.

|  | Our case | Anaya-Ayala et al. (3) | Okuda et al. (4) | Schneider et al. (7) | Berman et al. (8) |
| --- | --- | --- | --- | --- | --- |
| Age | 8 years | 11 years | 5 years | 3 years | 26 infants >8 weeks old |
| Presentation | Dyspnea, chest pain, non-bilious vomiting | Abdominal pain, nonproductive retching, cough, and shortness of breath | Cardiopulmonary arrest, pronounced dead on presentation | Dyspnea, fever, vomiting | Dyspnea, decreased feeds |
| Investigations | Chest x-ray and barium swallow | Chest x-ray | Post-mortem CT scan | Chest x-ray and barium swallow | Chest x-ray |
| Treatment | NG tube insertion and laparotomy | Thoracoscopic decompression and laparotomy | - | Chest tube and surgical repair | NG tube insertion and surgical repair |

# Supplementary Table

|  | Our case | Horst et al. (9) | Synder et al. (14) | Bunya et al. (16) | Ekim et al. (17) |
| --- | --- | --- | --- | --- | --- |
| Age | 8 years | 4 children - 3, 4, 6, and 13 months | 29 months | 75 years | 31 years |
| Presentation | Dyspnea, chest pain, non-bilious vomiting | Progressive respiratory distress | Acute respiratory failure | Motor vehicle accident → cardiac arrest | Motor vehicle accident → severe respiratory distress and hypotension |
| Investigation | Chest x-ray and barium swallow | Chest x-ray and thoracoscopy | Chest x-ray | - | Chest x-ray and CT scan |
| Treatment | NG tube insertion and laparotomy | NG tube insertion and surgical repair | Tube thoracostomy, NG tube insertion and surgical repair | Resuscitative thoracotomy | Chest tube, thoracotomy and NG tube insertion. |

**Supplementary Table 1.** The tables show comparison between our case and the referenced cases in the discussion of our manuscript in terms of age, presentation, investigations, and treatment. The second table is the continuation of the cases. References for these cases are included in the brackets.
